# Harder, Better, Faster, Stronger? Residents Seeing More Patients Per Hour See Lower Complexity

**DOI:** 10.5811/westjem.20282

**Published:** 2025-01-31

**Authors:** Corlin M. Jewell, Guangyu (Anthony) Bai, Dann J. Hekman, Adam M. Nicholson, Michael R. Lasarev, Roxana Alexandridis, Benjamin H. Schnapp

**Affiliations:** * University of Wisconsin School of Medicine and Public Health, BerbeeWalsh Department of Emergency Medicine, Madison, Wisconsin; † Indiana University School of Medicine-Northwest, Gary, Indiana; ‡ University of Wisconsin School of Medicine and Public Health, Department of Biostatistics and Medical Informatics, Madison, Wisconsin

## Abstract

**Introduction:**

Patients seen per hour (PPH) is a popular metric for emergency medicine (EM) resident efficiency, although it is likely insufficient for encapsulating overall efficiency. In this study we explored the relationship between higher patient complexity, acuity on shift, and markers of clinical efficiency.

**Methods:**

We performed a retrospective analysis using electronic health record data of the patients seen by EM residents during their final year of training who graduated between 2017–2020 at a single, urban, academic hospital. We compared the number of PPH seen during the third (final) year to patient acuity (Emergency Severity Index), complexity (Current Procedural Terminology codes [CPT]), propensity for admissions, and generated relative value units (RVU).

**Results:**

A total of 46 residents were included in the analysis, representing 178,037 total cases. The number of PPH increased from first to second year of residency and fell slightly during the third year of residency. Overall, for each 50% increase in the odds of treating a patient requiring high-level evaluation and management (CPT code 99215), there was a 7.4% decrease in mean PPH. Each 50% increase in odds of treating a case requiring hospital admission was associated with a 6.7% reduction (95% confidence interval [CI] 0.73–12%; P = 0.03) in mean PPH. Each 0.1-point increase in PPH was associated with a 262 (95% CI 157–367; P < 0.001) unit increase in average RVUs generated.

**Conclusion:**

Seeing a greater number of patients per hour was associated with a lower volume of complex patients and patients requiring admission among EM residents.

Population Health Research Capsule What do we already know about this issue?

*Patients seen per hour (PPH) is commonly used by programs to measure efficiency in residents. It is unclear whether this adequately encapsulates efficiency.*

What was the research question?

*Can the use of multiple clinical metrics allow programs to better contextualize the meaning of resident efficiency in the ED?*

What was the major finding of the study?

*For each 50% increase in the odds of treating a high-complex case, there was a 7.4% (0.79–13.6%; P = 0.03) decrease in mean PPH.*

How does this improve population health?

*Residents who see more PPH may not treat as many complex patients, which could have implications for their readiness for independent practice.*

## INTRODUCTION

The 2019 American Board of Emergency Medicine Model of Clinical Practice recognizes task- switching and multiple patient care as core physician tasks,^1^ and the Accreditation Council for Graduate Medical Education (ACGME) lists multitasking as Emergency Medicine Patient Care Milestone 7.^2^ Emergency physicians (EP) must efficiently evaluate and treat a high volume of patients to effectively manage care in the emergency department (ED). Various metrics have been used to evaluate efficiency and quality of care provided in the ED by the ED staff as well as individual EPs (patient length of stay, ED admission rate, etc).^3^ ^,^ ^4^ A metric commonly used by programs to measure efficiency in residents is the number of patients seen per hour (PPH). This metric is enticing because it is based on data that is easily retrievable and widely applicable across clinical sites.^5^ ^,^ ^6^ However, it is currently unclear whether the number of PPH can adequately encapsulate efficiency in physician trainees. It is also uncertain how residency programs should consider this metric when assessing their trainees, especially if not considered alongside other metrics.

A physician-in-training who sees more PPH could potentially be seen as more capable of independently managing the higher number of patients required for independent practice. This measurement is already commonly used when evaluating EM residents and is also frequently used to evaluate attending EPs.^5^ ^,^ ^6^ However, it is unclear whether there are tradeoffs for residents that come with seeing a higher patient volume. It is likely that medical trainees are only able to handle a finite number of cognitive tasks before their performance is impaired and they are unable to take on additional tasks.

One method to conceptualize the relationship between how patient complexity and acuity impacts other aspects of patient care is through cognitive load theory.^7^ In general, when cognitive load is too high, such as increased extraneous load from managing multiple patients or increased intrinsic load from managing very complex patients, overall cognitive performance may be impaired. This could decrease cognitive bandwidth for new patient-care tasks as well as limit germane load to allow for learning and illness-scheme creation.^7^ Conversely, simple, straightforward patient presentations may not impose such a significant cognitive load, allowing cognitive resources to be deployed to see a higher volume of patients.^8^ ^,^ ^9^ Prior studies have assessed resident efficiency in the ED in terms number of PPH as training progresses.^10^ These studies have demonstrated that senior residents can see higher numbers of patients per hour compared to postgraduate year (PGY)-1 residents, which plateaus in the final year of training.^11^

Compared to advanced practice practitioners (APP) (physician assistants [PA] or nurse practitioners), residents see fewer PPH but generate a higher amount of relative value units (RVU). This suggests residents may see higher acuity patients or document more thoroughly.^10^ The RVUs are an objective means of measuring the resources needed to provide medical care as a single metric.^12^ Another means of estimating the resources needed to provide care are ED evaluation and management (E/M) Current Procedural Terminology (CPT) codes. These allow coders to use complexity in documentation as a surrogate marker of complexity of care provided. While RVUs and CPT codes are measures assigned following a patient’s ED encounter, the Emergency Severity Index (ESI) is a means of estimating the acuity of the patient in terms of priority and resources allocation based on their initial presentation.

It is currently unknown how patient complexity and acuity may impact markers of clinical efficiency for ED residents. Our aim in this study was to better evaluate this relationship using multiple metrics to allow residency leaders to better contextualize greater resident efficiency in the ED.

## METHODS

### Study Setting

The study was conducted at a single three-year EM residency program associated with an urban, academic ED located in the Midwestern US. The hospital in which the ED is situated is a Level I adult and pediatric trauma, burn, stroke, and STEMI center. The ED has 43 adult beds and sees approximately 60,000 patient visits per year. During the study period, the residency had 12 PGY-1 positions each year.

The adult ED is divided into three separate treatment areas with two primary treatment teams. Each treatment team consists of a single attending physicians as well as 2–3 PAs or resident physicians. Shifts are nine hours in duration. Throughout most of the study period, patients were treated by the team of physicians designated to that treatment area. In 2019, the ED shifted to a model in which either treatment team could care for any patient in either treatment area. Each treatment team is staffed by residents of any PGY level with at least one senior resident (PGY-2 or PGY-3). All residents were encouraged to assign themselves to patients of any acuity level. During the study, PAs were employed in the ED and could take the place of a resident on shift (especially during weekly resident didactics). The APPs had no additional restrictions or privileges compared to residents in assigning themselves to patients.

As staffing is variable, there are no specific number of patients that each resident is required to see per shift. All residents staff directly with the attending; no residents supervise other residents. During expected peak times (of patient arrival), a triage team consisting of a single attending physician and a PA is also present and generally sees the lowest acuity patients; all residents are assigned approximately the same number of shifts but may freely trade shifts among themselves. While attending physicians can assign themselves to patients primarily (ie, no resident or APP assigned), this is a rare occurrence and typically occurs only during times of excessive patient volume or acuity.

### Study Design and Population

We designed this study as a retrospective observational study using aggregated, resident case data extracted from the electronic health record (EHR) (Epic Systems, Verona, WI). Data for PGY 1–3 residents were extracted for four consecutive classes of residents who graduated between 2017–2020. To remove significant outliers we excluded residents if they did not graduate from the program within three consecutive years. We collected data on the characteristics of the patients seen as well as markers of residency efficiency for all available patient encounters during the study period (Table 1). Multiple metrics were used to provide a more accurate measure of patient complexity rather than a single metric in isolation. The research team was composed of a senior resident (TB) and a departmental data analyst (DH), as well as faculty educators (CJ, AN, BS). We chose the selected markers as they have been used as markers of resident clinical efficiency in other studies.^6^ ^,^ ^10^

**Table 1.** Emergency medicine resident metrics of efficiency and the characteristics of patients seen.

Metric	Description
Patient characteristics	
Emergency Severity Index (ESI)	Frequency of patient encounters matching each ESI score (1–5). This is a means of estimating time and resource allocation for a patient based on their initial presentation.
Evaluation and management (E/M) Current Procedural Terminology (CPT) codes	Frequency of patient encounters receiving each E/M CPT code (99281–99285). These represent a means of determining patient complexity based on meeting certain documentation criteria.
Hospital admission	Number of patient encounters in which an inpatient admission occurred
Efficiency metrics	
Relative value units (RVU)	Total number of work RVUs generated
Patients seen per hour	Total number of patients seen divided by total number of hours worked in the ED during PGY-3

*ED*, emergency department; *PGY*, postgraduate year.

Patient care was attributed to the first assigned resident, as this resident is typically the most cognitively and practically involved in the patient’s care. Patients who are signed out to an oncoming ED team are shared equally among all oncoming residents. We excluded pediatric patient encounters (ie, patients <18 years of age) as pediatric cases have substantial differences in terms of the resources and cognitive load required to provide adequate care. Therefore, it was determined that the chosen efficiency metrics could not be meaningfully compared to adult patient encounters.^13^ For example, the average length of stay between pediatric and adult encounters during the study period was 219 vs 362 minutes. Over the course of their training, residents complete a dedicated block of pediatric ED shifts during their first and second years and complete an additional 1–3 pediatric ED shifts during each adult ED rotation. We calculated the percentage of patient encounters compared to overall patient encounters.

Given the aggregated nature of the data that did not contain any patient protected health information or identifying resident data, no informed consent was collected. The data was extracted from the EHR by the departmental data analyst and was stored on a password-protected departmental server available only to members of the study team. No additional chart review was conducted on the included encounters. This study was determined to be quality improvement and exempt from formal review by our institutional review board.

### Statistical Analysis

We calculated the PPH for each PGY-3 resident by using the total number of adult patient encounters for which they were the first resident assigned, divided by the total number of hours worked in the adult section of the ED. Residents were grouped based on the year of graduation. A two-sided significance level of *P* < 0.05 was used for all statistical tests. We performed all statistical analyses and graphics using R version 4.1.1 (R Core Team, R Foundation for Statistical Computing, Vienna, Austria). We used negative binomial regression to assess the relationship between PPH and the odds of treating a patient who required admission, adjusted for hours worked and patient complexity. All analyses were performed at the resident level.

To determine the relationship between ESI and PPH, we first dichotomized ESI into high and low severity. High severity included encounters from the third year of residency that were labeled ESI 1 and 2 and low severity included encounters that were labeled ESI 3, 4, and 5. The ESI 1 encounters were not separately analyzed as these are relatively rare compared to the overall number of patient encounters. We then calculated the odds of treating a patient with a high-severity ESI. The relationship between CPT codes and PPH was similarly calculated by dichotomizing CPT into more and less complex. More complex included the highest complexity CPT code (99285), and less complex included the remaining four codes (99281–99284). We did not consider CPT code 99291 as only attendings can bill for critical care, and there is significant variation within our attending group in the use of critical care billing. Therefore, we believed that this was less likely to be a resident-sensitive metric. We similarly calculated the odds of treating a patient with a more complex CPT. To assess significant differences among PGY that could introduce bias, we used the Kruskal-Wallis test and the Nemenyi procedure for post-hoc comparisons.^14^

We used RVUs as a proxy for shift complexity and regressed that as the response in a multivariable regression model using PPH, PGY, and the interaction between PPH and PGY as explanatory variables.

## RESULTS

A total of 46 residents met inclusion criteria. One resident was excluded who had a non-consecutive training period, and another resident left the program prior to graduation at the end of their PGY-1 year. Overall, 1.6% of the total patient encounters were assigned 99291/99292 CPT codes and were excluded from that analysis. An additional 17.6% of total patient encounters, consisting of pediatric cases, were also excluded, leaving a total of 178,037 patient encounters. Average PPH data for the four included PGYs can be seen in Table 2. The average ESI during the study period was 2.8.

**Table 2.** Patients seen per hour data for class years 2017–2020.

Class year	Academic year	Mean PPH (95% CI)
2017	2014–2015 PGY-1	1.20 (1.13–1.28)
	2015–2016 PGY-2	1.51 (1.42–1.61)
	2016–2017 PGY-3	1.52 (1.43–1.62)
2018	2015–2016 PGY-1	1.11 (1.05–1.16)
	2016–2017 PGY-2	1.50 (1.43–1.58)
	2017–2018 PGY-3	1.45 (1.39–1.52)
2019	2016–2017 PGY-1	1.08 (1.03–1.13)
	2017–2018 PGY-2	1.37 (1.31–1.44)
	2018–2019 PGY-3	1.26 (1.21–1.32)
2020	2017–2018 PGY-1	1.01 (0.96–1.05)
	2018–2019 PGY-2	1.33 (1.28–1.39)
	2019–2020^*^ PGY-3	1.09 (1.04–1.14)

* May have been impacted by the COVID-19 pandemic.

*CI,* confidence interval;* PPH*, patients seen per hour; *PGY*, postgraduate year.

### Current Procedural Terminology

**Figure 1.**
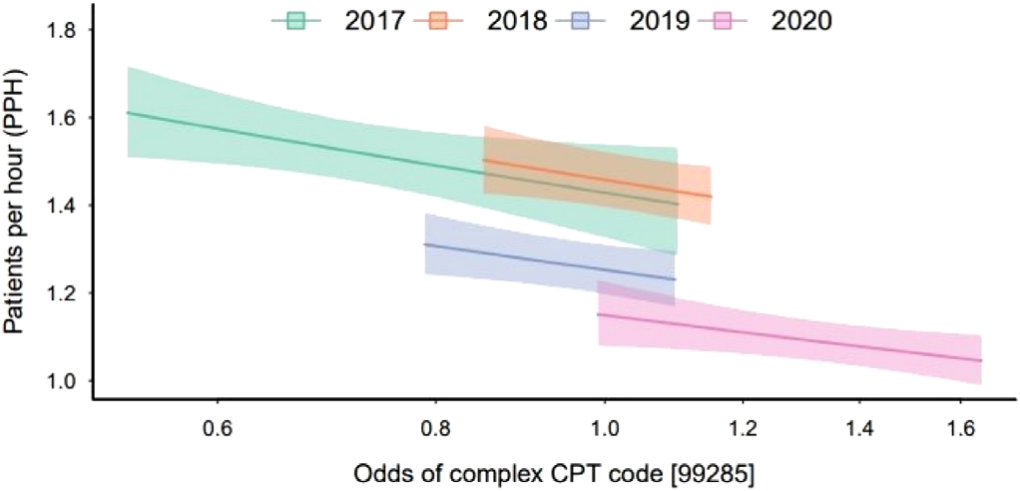
Relationship between odds of treating a high-complex case and mean patients seen per hour during postgraduate year-3, grouped by graduation year. Shaded regions represent 95% confidence intervals. *CPT*, Current Procedural Terminology.

Adjusted for class year, a 50% increase in the odds of treating a complex case was associated with the mean PPH decreasing 7.42% (95% confidence interval [CI] 0.79–13.6% reduction in mean PPH; *P* = 0.03). The relationship between PPH and odds of treating a high-complexity case can be seen in Figure 1.

### Hospital Admission

**Figure 2.**
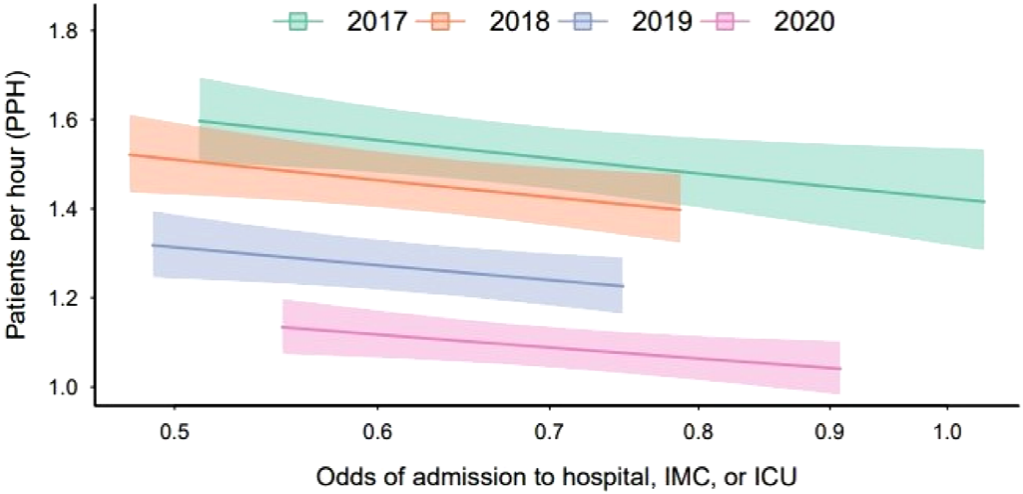
Relationship between odds of a case resulting in admission and mean patients seen per hour during postgraduate year 3, grouped by graduation year. Shaded regions represent 95% confidence intervals. *IMC*, intermediate care unit; *ICU*, intensive care unit.

Each 50% increase in odds of treating a case requiring hospital or intensive care unit [ICU]/intermediate care unit admission was associated with a 6.7% (95% CI 0.73–12%; *P* = 0.03) reduction in mean PPH. The relationship between PPH and odds of treating a case requiring admission can be seen in Figure 2.

### Emergency Severity Index

After controlling for PGY, there was no significant relationship observed between PPH and the odds of treating a high acuity case (*P* = 0.30).

### Relative Value Units

**Figure 3.**
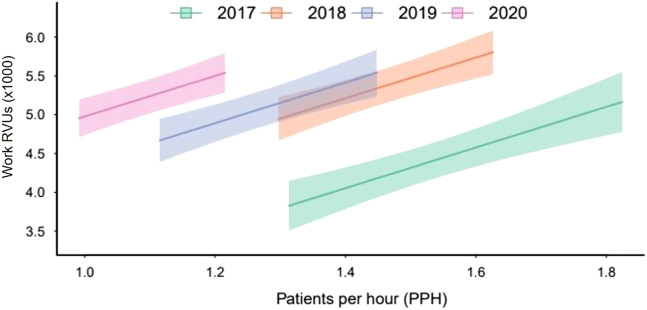
The relationship between relative value units generated and patients seen per hour during postgraduate year 3, grouped by graduation year. Shaded regions represent 95% confidence intervals. *RVU*, relative value units.

The models suggested that each 0.1 point increase in PPH is associated with a 262 unit increase (95% CI 157–367; *P* < 0.001) in average work RVUs generated, with the association between average total RVU and PPH stable across the four years. See Figure 3 for the relationship between RVUs generated and PPH.

## DISCUSSION

Residents seeing higher numbers of patients saw fewer complex patients and fewer patients requiring an inpatient admission. We believe this study is the first to examine the association of patient complexity and acuity on the clinical efficiency with which EM residents operate. As suggested by cognitive load theory, we found that residents’ capacity to pick up complex patients in this study was finite. More complex patients and patients requiring admission may impose more of a task load (eg, phone calls to consultants or admitting physicians, review of records, or longer history-taking) than patients with lower acuity. This greater cognitive load could result in a decrease in PPH as complexity goes up. This effect may be mitigated somewhat by a variety of effective clinical practices, such as partnering with nurses or assistance from their supervising attending. However, more research is needed to determine whether other factors, such as the incorporation of evidence-based efficiency practices or adding scribes for documentation, may affect resident efficiency.

Our data shows that PPH rises sharply between PGY-1 and -2 years and then plateaus between the PGY-2 and -3 years. This finding is in line with previous literature.^11^ While the underlying cause of this finding is ultimately unknown, it may be secondary to changes in focus that occur between the latter years in training. For example, any further increases in the ability of PGY-3 residents to see additional patients over a PGY-2 resident may be offset by a focus on departmental flow, instruction of junior learners, or simply succumbing to “senioritis.” It is also possible that the most senior residents preferentially selected the most critically ill patients in the ED and the increased complexity of these patients were the reason for the plateau.

We found no significant relationship between PPH and ESI. However, there was a negative relationship when evaluating PPH and CPT codes as well as the likelihood of caring for a patient who would need to be admitted. This may be because ESI is assigned at the beginning of the patient’s treatment course, whereas CPT designation and admission decisions are made later in the patient’s course (or after the conclusion of the encounter in the case of CPT). The ESI was also treated as a binary variable for analysis, with ESI 3 treated as a low-acuity patient. However, many of these patients may have a higher acuity illness; it is possible that this dichotomization eliminated a true effect that would otherwise have been seen. Therefore, it could reflect that ESI could not be used to accurately estimate the amount of resources and cognitive effort required to care for these patients.^15^

While we did not analyze the relationship between patient complexity and overall generation of RVUs, it remains an interesting avenue for future research. While it makes intuitive sense that the care of a single, more complex patient would generate more RVUs than a single, less complex patient, it is unknown whether RVU generation is balanced by the increased amount of time and cognitive load these patients often require. This was not done in the current study as this would also have depended on hospital crowding, which is a confounding variable we chose not to include.

Overall, our results suggest that the use of PPH as a surrogate measure of patient efficiency may paint an incomplete picture of resident performance. While the current study did demonstrate a statistically significant relationship between patient complexity and PPH, the clinical significance is unclear. The required number of patients seen during training represents a critically unexplored area of residency training. Experiential learning theory would suggest that seeing a greater number of patients would result in a higher level of competence, but this may be mediated by complexity or other factors. Residency leadership teams who plan to evaluate their residents on their ability to task switch between multiple patients (ACGME Milestone PC7) may wish to explore the use of other markers that may correlate with PPH. These may better capture the complexity of the care provided, although further study is required before this can be considered best practice.

## LIMITATIONS

An important limitation of this study is its single-center design. The results seen may be due to unique factors of the study site and, therefore, may not be generalizable to other sites. For example, the study site changed from a pod-based model in 2019, which may have restricted the efficiency of some residents, to a “free-for-all” model where residents could assign themselves to new patients as soon as they were ready. Additionally, there may have been subtle changes to the patient population seen by the residents over the years, or changes to the residency, that were not assessed in the current study. For example, the final year of the study data included a few months that were affected by the COVID-19 pandemic. This would only have impacted a small portion of the final year of training for the Class of 2020. However, it may have led to the discrepancy seen in PPH between the Class of 2020 and the other included classes as seen in Figure 3. It is interesting that this did not result in a substantial change in RVUs generated. No specific documentation interventions were implemented during this time and may simply represent general changes in documentation practices.

We did not factor in how patients who were taken in sign-out would affect the utilized metrics. It is likely that residents who were signed out patients requiring multiple additional actions (such as consultation calls, procedures, etc) would negatively impact their ability to take on new patients. These cases were excluded because it would have been unfeasible to account for how much additional work was required for these patients. For example, some patients, even those who were critically ill, may be signed out when all major diagnostic and therapeutic interventions have already been completed, and the patient is simply awaiting transfer to the hospital floor.

We did not consider patients who were specifically admitted to our step-down ICU units, or those who went directly to the operating room. While the rate of admission to these locations could certainly imply a level of complexity, the way this is determined varies greatly between institutions and would have added a significant layer of complexity to the current study. At our institution, we have two affiliated hospitals that we can admit patients to, each with different levels of capabilities and different criteria for ICU/stepdown unit status. This represents an interesting avenue of future study.

We also excluded patients assigned CPT codes 99291 and 99292 (which denote critical care) from our analysis of the relationship between PPH and CPT codes. This was done as critical care billing can only be done by the attending physician, and documentation practices for this are variable within our attending group. The overall percentage of patients who received 99291 or 99292 CPT codes was only 6%. However, these patients were not excluded entirely and would have been included in the analysis of other metrics apart from CPT. As stated earlier, the use of multiple metrics in this study was designed to overcome limitations in individual metrics alone.

It is possible that the presence of triage physician during peak hours of patient arrival may have impacted the metrics used in this study. While this was not specifically controlled for, the triage physician team primarily sees only the lowest acuity patients (eg, simple laceration repairs, ankle sprains, needlestick injuries) and was felt to not have a big impact on our chosen metrics. We did not wish to exclude shifts in which the triage physician was present as this timeframe represents the highest patient census in our ED. If an impact occurred, this would be expected to decrease the magnitude of the relationship between PPH and the chosen variables. Despite this, a significant effect was still demonstrated.

Finally, this numerical data does not completely encapsulate other factors that would influence a resident’s overall efficiency. These factors could include their clinical abilities and medical knowledge. Because of this, we caution residency programs from looking at the variables investigated in this study in isolation when assessing their own trainees.

## CONCLUSION

Residents caring for higher numbers of patients per hour were associated with fewer complex patients and patients who required inpatient admission.

## Supplementary Information




