# Supplementary material for: Harder, Better, Faster, Stronger? Residents Seeing More Patients Per Hour See Lower Complexity
Source: West J Emerg Med. 2025 Jan 31;26(2):254–60. doi: 10.5811/westjem.20282 (PMC11931708; doi:10.5811/westjem.20282)
Supplement: Supplementary file 1 [file wjem-26-254-s001.docx]

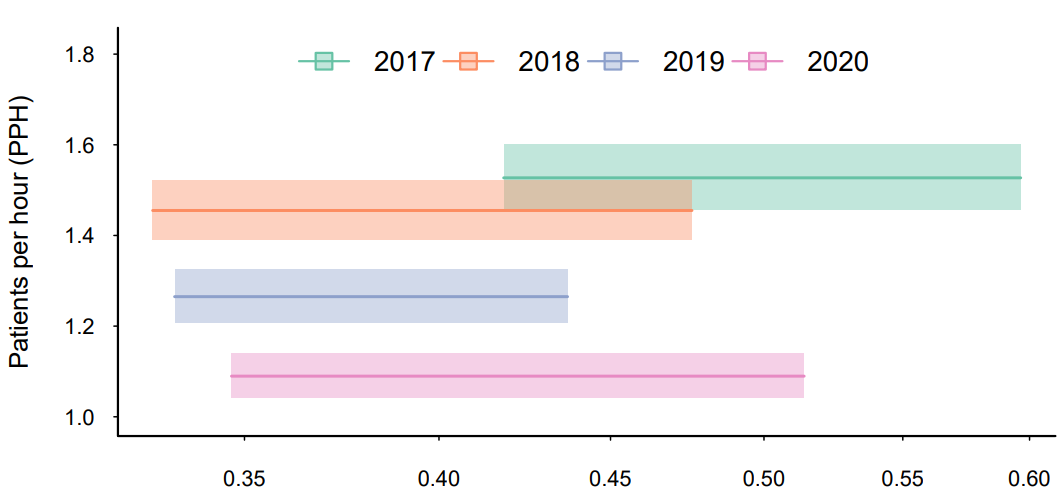


**Figure S1. Relationship between Odds of a High Acuity Case (ESI 1 or 2) and Mean PPH during PGY-3 year, grouped by graduation year. Shaded regions represent 95% confidence intervals.**
